# Serum cortisol predicts death and critical disease independently of CRB-65 score in community-acquired pneumonia: a prospective observational cohort study

**DOI:** 10.1186/1471-2334-12-90

**Published:** 2012-04-13

**Authors:** Martin Kolditz, Gert Höffken, Peter Martus, Gernot Rohde, Hartwig Schütte, Robert Bals, Norbert Suttorp, Mathias W Pletz

**Affiliations:** 1 Division of Pulmonology, Medical Department 1, University Hospital Carl Gustav Carus, Fetscherstr. 74, 01307 Dresden, Germany; 2 Department of Biostatistics and Clinical Epidemiology, Charité Berlin, Germany; 3 Department of Respiratory Medicine, Maastricht University Medical Center, Maastricht, The Netherlands; 4 Department of Internal Medicine, Infectious Diseases and Pulmonary Medicine, Charité - Universitätsmedizin Berlin, Berlin, Germany; 5 Department of Internal Medicine V - Pulmonology, Allergology, Respiratory Intensive Care Medicine, Saarland University Hospital, Homburg, Germany; 6 Division of Gastroenterology, Hepatology and Infectious Diseases, Department of Internal Medicine II, Jena University Hospital, Jena, Germany

**Keywords:** Risk stratification, Prognosis, Biomarker, Mortality, CRB-65 score

## Abstract

**Background:**

Several biomarkers and prognostic scores have been evaluated to predict prognosis in community-acquired pneumonia (CAP). Optimal risk stratification remains to be evaluated. The aim of this study was to evaluate serum cortisol as biomarker for the prediction of adverse outcomes independently of the CRB-65 score und inflammatory biomarkers in a large cohort of hospitalised patients with CAP.

**Methods:**

984 hospitalised CAP-patients were included. Serum cortisol was measured and its prognostic accuracy compared to the CRB-65 score, leucocyte count and C-reactive protein. Predefined endpoints were 30-day mortality and the combined endpoint critical pneumonia, defined as presence of death occurring during antibiotic treatment, mechanical ventilation, catecholamine treatment or ICU admission.

**Results:**

64 patients died (6.5%) and 85 developed critical pneumonia (8.6%). Cortisol levels were significantly elevated in both adverse outcomes (p < 0.001) and predicted mortality (AUC 0.70, cut-off 795 nmol/L) and critical pneumonia (AUC 0.71) independently of all other measured variables after logistic regression analysis (p = 0.005 and 0.001, respectively). Prognostic accuracy of CRB-65 was significantly improved by adding cortisol levels (combined AUC 0.81 for both endpoints). In Kaplan-Meier analysis, cortisol predicted survival within different CRB-65 strata (p = 0.003). In subgroup analyses, cortisol independently predicted critical pneumonia when compared to procalcitonin, the CURB65 score and minor criteria for severe pneumonia according to the 2007 ATS/IDSA-guideline.

**Conclusion:**

Cortisol predicts mortality and critical disease in hospitalised CAP-patients independently of clinical scores and inflammatory biomarkers. It should be incorporated into trials assessing optimal combinations of clinical criteria and biomarkers to improve initial high risk prediction in CAP.

## Background

A disease severity based approach with initial risk stratification is required to guide management and treatment decisions in CAP. International guidelines recommend prognostic scores to support clinical decision on management as outpatient, inpatient or admission to ICU. The most established score systems are the PSI-score and the CURB or CRB-65 scores, which perform comparably for mortality prediction and identification of low risk patients suitable for an outpatient management strategy [1-3]. Amongst them, the CRB-65 score is the most easily to calculate and widely used in Europe [4,5].

Additionally, biomarkers have been found to improve risk stratification and management decisions in CAP. Procalcitonin (PCT) has been demonstrated to provide additional information by identifying low risk patients [6], as parameter to judge treatment response when sequentially measured during treatment [7] and as a tool to guide antibiotic treatment duration [8]. Other biomarkers like pro-ADM, pro-ANP, pro-BNP, pro-vasopressin and d-dimer have been associated with mortality in CAP [9-13].

However, accurate mortality prediction does not automatically lead to accurate identification of patients developing critical disease in need for intensive care treatment [14]. Both the CRB-65 and PSI scores lack accuracy for the prediction of high risk situations resulting in ICU admission [14]. Other scoring systems have been proposed to identify patients requiring admission to ICU like the modified American Thoracic Society (ATS)-rule [15] and more recently the new ATS/Infectious Diseases Society of America (IDSA)-recommended score [16]. However, both scores still fail to identify a significant proportion of patients with early deterioration and have poor positive predictive values for ICU-admission [17-19]. Thus, identifying patients with a high benefit from initial intensive management strategies in CAP remains an important task to be done [20].

Recently, serum cortisol concentration was shown to be associated with severity and mortality in CAP in three small trials [21-23]. Additionally, we demonstrated that cortisol predicts persistent clinical instability, making it a potential parameter to improve the identification of patients with high risk for a complicated disease course [21].

The aim of this trial was, to evaluate serum cortisol as biomarker for the development of severe disease requiring more intensive monitoring and management strategies as well as 30-day mortality in a large cohort of hospitalised CAP-patients. Additionally, we studied the independent contribution of serum cortisol to the prognostic properties of the CRB-65 score and inflammatory biomarkers.

## Methods

### Patients

Hospitalised CAP patients were recruited from a multicentre national CAP-study in Germany, the German Competence network for the study of CAP (CAPNETZ). Detailed description of the CAPNETZ methodology is given elsewhere [24]. Inclusion criteria for entering the study are: age ≥ 18 years, pulmonary infiltrate diagnosed by chest x-ray, clinical symptoms consisting of cough or purulent sputum or positive auscultation or fever. Among the exclusion criteria is systemic steroid medication of ≥ 20 mg prednisolone equivalent per day for more than 14 days. Patients for this study were recruited in 12 clinical CAPNETZ centers between 10/2002 and 12/2008. All clinical parameters are stored in an electronic database. Written informed consent was obtained from every patient prior to inclusion in the study. The study was approved by the ethical review board of the University of Magdeburg, Germany (#104/01).

Comorbidities in the present study were defined as presence of one or more of the following: congestive heart failure, COPD, chronic renal disease, chronic liver disease, cerebrovascular disease, malignancy, or diabetes mellitus.

The CRB-65 score was determined in all patients as described previously [25].

All patients were followed up according to a standardized data sheet for at least 180 days.

### Laboratory values

Venous blood samples were collected within 24 h after first presentation and inclusion in the CAPNETZ study and stored centrally at -70°C. Leucocyte (WBC) count and urea were determined by the local hospital laboratories. Serum C-reactive protein (CRP) was centrally measured by nephelometry with a commercially available assay (Behring Diagnostics, Marburg, Germany). Serum PCT was centrally determined by an immunofluorescent assay (sensitive KRYPTOR, B.R.A.H.M.S AG, Henningsdorf, Germany). Serum cortisol was centrally analysed by a commercially available chemiluminescence immunoassay (Roche Diagnostics, Mannheim, Germany). This assay has a measurement range of 0.5-1750 nmol/L, higher concentrations were analysed by dilution of the serum sample according to the manufacturers protocol.

### Outcome parameter

Predefined outcome variables were survival within 30 days after first contact and the combined endpoint critical pneumonia, defined as the presence of one of the following: death occurring during antibiotic treatment, mechanical ventilation, catecholamine treatment or ICU admission.

### Statistics

Two group comparisons were calculated by the Mann-Whitney *U*-test. Univariate and multivariate analyses were performed to predict the binary endpoints by including the CRB-65-score, the measured laboratory parameters WBC, CRP, cortisol, and all documented comorbid conditions and demographic factors given in Table 1. For multivariate analyses a logistic regression model with stepwise forward variable selection was employed.

**Table 1:** Baseline characteristics of patients

characteristic	Total (n = 984)	**Non-survivors **^**a**^ (N = 64)	**Survivors **^**a**^ (N = 920)	**p-value **^**b**^	**Critical CAP **^**c**^ (N = 85)	**No critical CAP **^**c**^ (N = 899)	**p-value **^**b**^
**Age **(years ± SD)	58.7 (± 18.1)	75.4 (± 9.8)	57,5 (± 17.9)	**< 0.001**	69.5 (± 13.4)	57.7 (± 18.1)	**< 0.001**
**Male sex **N (%)	558 (56.7)	46 (71.8)	512 (55.7)	**0.011**	59 (69)	499 (56)	**0.013**
**Smoker **N (%)	309 (31.4)	15 (23.4)	294 (32.0)	0.15	28 (32.9)	281 (31.3)	0.75
**Prior antibiotics **N (%)	276 (28.0)	12 (18.8)	264 (28.7)	0.09	15 (17.6)	261 (29.0)	**0.026**
**Nursing home **N (%)	28 (2.8)	7 (10.9)	21 (2.3)	**< 0.001**	10 (11.8)	18 (2.0)	**< 0.001**
**Pathogen established **N(%)	195 (19,8)	13 (20,3)	182 (19,8)	0.92	23 (27,1)	172 (19,1)	0.08
**Comorbidities **N (%)							
Congestive heart failure	150 (15.2)	29 (45.3)	121 (13.2)	**< 0.001**	31 (36.5)	119 (13.3)	**< 0.001**
Chronic respiratory disease	360 (36.6)	27 (42.9)	333 (36.4)	0.30	41 (48.8)	319 (35.6)	**0.017**
Chronic renal disease	71 (7.2)	17 (27.0)	54 (5.9)	**< 0.001**	14 (16.7)	57 (6.4)	**< 0.001**
Chronic liver disease	33 (3.4)	2 (3.1)	31 (3.4)	0.91	0 (0)	33 (3.7)	0.07
Cerebrovascular disease	60 (6.1)	11 (17.2)	49 (5.3)	**< 0.001**	11 (12.9)	49 (5.5)	**0.006**
Malignancy	88 (8.9)	16 (25.0)	72 (7.9)	**< 0.001**	13 (15.3)	75 (8.4)	**0.034**
Diabetes mellitus	164 (16.7)	20 (31.3)	144 (15.7)	**0.001**	27 (31.8)	137 (15.2)	**< 0.001**
**CRB-65 **N (%)				**< 0.001**			**< 0.001**
0	417 (42.4)	3 (4.7)	414 (45.0)		9 (10.6)	408 (45.4)	
1	415 (42.2)	32 (50)	383 (41.6)		31 (36.5)	384 (42.7)	
2	132 (13.4)	24 (37.5)	108 (11.7)		34 (40.0)	98 (10.9)	
3	19 (1.9)	4 (6.3)	15 (1.6)		10 (11.8)	9 (1)	
4	1 (0.1)	1 (1.6)	0 (0)		1 (1.2)	0 (0)	
**Biomarker **(median, IQR^d^)							
Cortisol (nmol/L)	621 (419-866)	870 (624-1359)	602 (407-843)	**< 0.001**	972 (626-1520)	598 (406-837)	**< 0.001**
WBC^e ^(10^9^/mL)	10.7 (8.0-14.5)	13.2 (9.4-16.8)	10.6 (8.0-14.3)	**0.003**	13.6 (10.2-17.1)	10.5 (7.9-14.3)	**< 0.001**
CRP^f ^(mg/L)	71.9 (18.8-167)	121.3 (55-230)	67.9 (18-163)	**< 0.001**	141 (73-246)	66 (17-158)	**< 0.001**

^a ^survival at 30 days; ^b ^bold values indicate significant differences; ^c ^critical CAP = presence of one of the following: death occurring during antibiotic treatment, mechanical ventilation, catecholamine treatment or ICU admission; ^d ^IQR = interquartile range; ^e ^WBC = leucocyte count; ^f ^CRP = C-reactive protein

Receiver operating characteristic (ROC) curve analysis was used to determine the diagnostic properties of predictive parameters, optimal cut-off values were determined by Youden's index. To compare the predictive value of cortisol and the CRB-65 score, a binary logistic regression model of both parameters was used and the predicted probability derived from that model was included into the ROC-curve analysis. Odds ratios were calculated by using the Mantel-Haenszel estimate.

To assess the influence of cortisol concentrations on mortality, we produced Kaplan-Meier survival curves according to cortisol quartiles or cut-off value and stratified by CRB-65 score. Comparison between the groups was performed by log-rank test. A p-value of ≤ 0.05 (two-sided) was considered statistically significant. Statistical analyses were performed with SPSS for Windows, version 17.0 (SPSS Inc., Chicago, IL, USA).

## Results

984 patients were included in the study, their baseline characteristics are summarised in Table 1.

A causative microbiological pathogen was detected in 195 patients (20%). Of these, typical bacteria were found in 84 patients (43%), atypical bacteria in 64 (33%), viruses in 29 (15%) and mixed infection in 18 (9%). *Streptococcus pneumoniae *was the most frequently isolated pathogen (n = 71, 36%)

There were 64 (6.5%) non-survivors at day 30 after the first contact; their characteristics are shown in Table 1. Mortality rates within the different CRB-65 score groups were 0.7% (0 points), 7.7% (1 point), 18.2% (2 points), 21.1% (3 points) and 100% (4 points). The predefined criteria for the combined endpoint of critical pneumonia occurred in 85 patients (8.6%): Death during antimicrobial treatment (N = 37), ICU-admission (N = 19, 2 died during antimicrobial treatment), mechanical ventilation (N = 44, 11 of them on ICU, 2 more died during antimicrobial treatment) or catecholamine treatment (N = 5, all on ICU).

**Figure 1:**
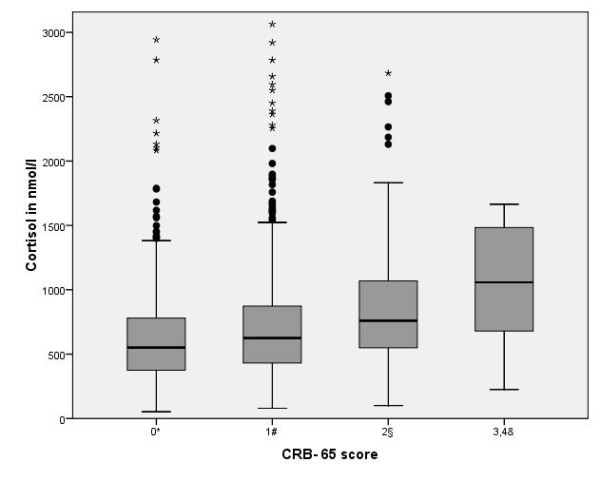
**Boxplots of cortisol levels by severity of CAP according to CRB-65 score**. * 1 case (cortisol 4509 nmol/l) not shown; # 3 cases (cortisol 3894-7817 nmol/l) not shown; § 2 cases (cortisol 4116, 9558 nmol/l) not shown; & 2 cases (cortisol 3858, 3924 nmol/l) not shown.

Serum cortisol levels on admission were significantly higher in non-survivors when compared to survivors (median 870 [IQR 624-1359] vs. 602 [407-843] nmol/l, p < 0.001) and in patients with critical pneumonia versus noncritical pneumonia (median 972 [626-1520] vs. 598 [406-837] nmol/l, p < 0.001, Table 1), and increased with increasing CRB-65 score (Figure 1). Cortisol values for the different criteria of critical pneumonia are shown in Table 2.

**Table 2:** Cortisol values for the different criteria of critical pneumonia

	cortisol (nmol/L; median, IQR^a^)		
Criteria	Yes	no	p-value
Death during antimicrobial treatment (N = 37)	1020 (634-1445)	607 (409-857)	< 0.001
ICU-admission (N = 19)	1013 (605-1831)	615 (414-866)	0.002
mechanical ventilation (N = 44)	985 (636-1648)	607 (409-853)	< 0.001
catecholamine treatment (N = 5)	1160 (391-1596)	621 (418-866)	0.25

^a ^IQR = interquartile range

In multivariate analysis including all variables given in Table 1, only cortisol level (p = 0.005 and 0.001 per IQR-increase, respectively), CRB-65 score, pre-existing congestive heart failure and male sex independently predicted both endpoints (Table 3).

**Table 3:** Factors predictive for the tested endpoints in multivariate logistic regression analysis, including all variables given in Table 1

	Mortality at 30 days		**Critical CAP **^**a**^	
Variable	OR (95% CI)	p-value	OR (95% CI)	p-value
Cortisol^b^	1.51 (1.13-2.01)	0.005	1.56 (1.20-2.02)	0.001
C-reactive protein^c^		0.1	1.45 (1.13-1.87)	0.004
CRB65	1.62 (1.07-2.47)	0.024	3.05 (2.22-4.19)	< 0.001
Congestive heart failure	2.20 (1.21-4.03)	0.010	1.96 (1.13-3.39)	0.017
Chronic renal disease	2.05 (1.03-4.04)	0.040		0.64
Malignancy	2.97 (1.50-5.89)	0.002		0.25
Age (years)	1.06 (1.03-1.09)	< 0.001		0.87
Male sex	2.13 (1.13-3.99)	0.019	1.73 (1.02-2.94)	0.043

^a ^critical CAP = presence of one of the following: death occurring during antibiotic treatment, mechanical ventilation, catecholamine treatment or ICU admission

^b ^cortisol levels encoded by quartiles in nmol/l: 1. quartile 52-420; 2. quartile 420-621; 3. quartile 621-866; 4. quartile 866-9558

^c ^C-reactive protein levels encoded by quartiles in mg/L: 1. quartile < 1-18.8; 2. quartile 18.8-71.9; 3. quartile 71.9-167; 4. quartile 167-578

This was confirmed by ROC-curve-analysis, showing that serum cortisol was associated with both predefined endpoints (AUC = 0.70 for 30-day mortality, AUC = 0.71 for critical pneumonia). The optimal cut-off value was calculated with 795 nmol/l with a sensitivity of 66% and specificity of 71% (OR 4.7 [95%-CI: 2.8-8.1], p < 0.001) for 30-day mortality. Sensitivity and specificity for critical pneumonia were 64% and 72% (OR 4.5 [95%-CI: 2.8-7.1], p < 0.001).

**Figure 2:**
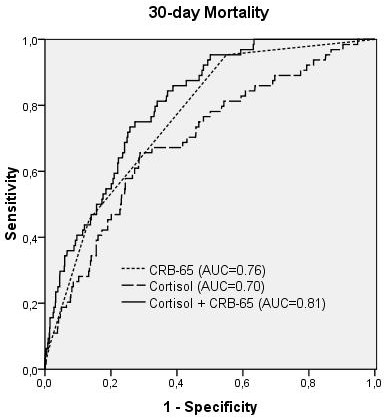
**ROC-Plot for 30-day mortality, comparing CRB-65 score, cortisol and CRB65-score plus cortisol (p = 0.001 versus CRB65 score alone)**.

**Figure 3:**
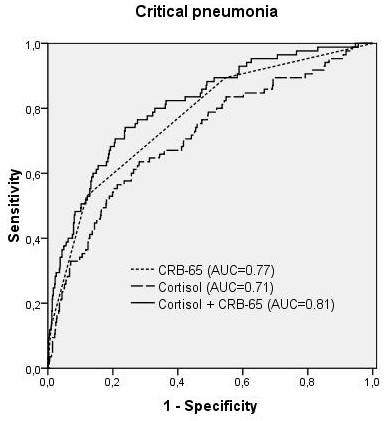
**ROC-Plots for 30-day critical pneumonia, comparing CRB-65 score, cortisol and CRB65-score plus cortisol (p = 0.002 versus CRB65 score alone)**.

As shown in binary logistic regression analysis, the combined use of CRB-65 and cortisol significantly improved the prediction of 30-day mortality (AUC = 0.81, p = 0.001) and critical pneumonia (AUC = 0.81, p = 0.002) when compared to the CRB-65 score alone (AUC = 0.76 and 0.77, respectively, Figures 2 and 3). This result remained significant, when CRP and WBC were added in the binary regression analysis (p = 0.006 and p = 0.018 for cortisol). WBC did not add significantly to the CRB-65 score for both endpoints, CRP improved prediction of critical pneumonia (combined CRP + CRB-65 AUC = 0.81, p = 0.004), but not 30-day mortality (p = 0.49).

**Figure 4:**
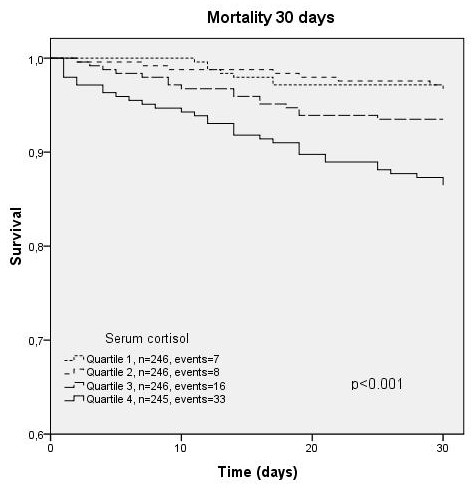
**Kaplan-Meyer analysis for 30-day mortality according to cortisol quartiles (2/984 cases censored before day 30)**.

**Figure 5:**
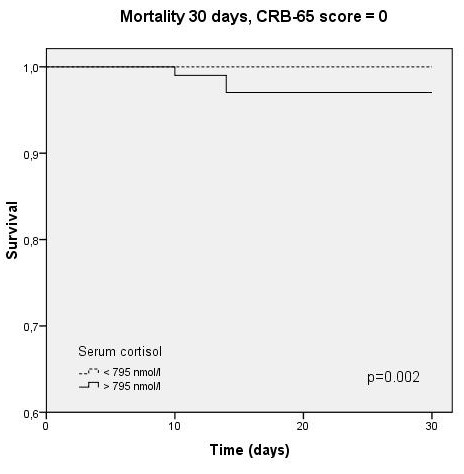
**Kaplan-Meyer analysis for 30-day mortality within CRB-65 score class 0 (2/984 cases censored before day 30)**.

**Figure 6:**
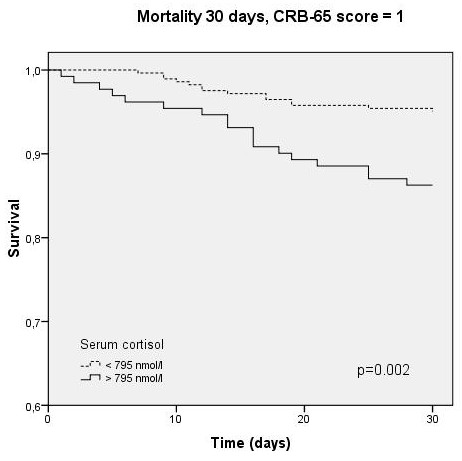
**Kaplan-Meyer analysis for 30-day mortality within CRB-65 score class 1 (2/984 cases censored before day 30)**.

**Figure 7:**
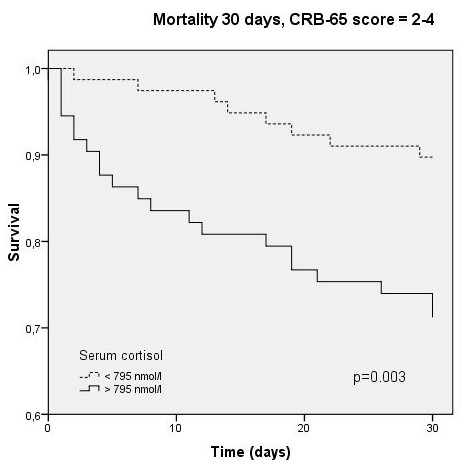
**Kaplan-Meyer analysis for 30-day mortality within CRB-65 score class 2-4 (2/984 cases censored before day 30)**.

Independent prediction of survival by cortisol could also be demonstrated by Kaplan-Meyer survival analysis. Survival was significantly different within cortisol-quartiles (p < 0.001, Figure 4), and this prediction persisted within the CRB-65 classes (p = 0.002-0.003, Figures 5, 6, 7).

Urea was available from 889 patients (90%), and in this subgroup a post-hoc analysis of the CURB- and CURB65-scores was performed. The CURB-score predicted 30-day mortality and critical pneumonia equally to the CRB-65 score with an AUC of 0.77 and 0.78, respectively. The corresponding values for the CURB-65 score were 0.81 and 0.79, which was a superior to the CRB65 score (p = 0.001). If cortisol was added, further significant improvement of prediction resulted for both scores and endpoints (cortisol + CURB: AUC 0.81 and 0.82; p = 0.009 and < 0.001; cortisol + CURB65: AUC 0.84 and 0.82; p = 0.02 and < 0.001, respectively).

PCT was not routinely measured in this trial. However, in 643 patients (65.3%) initial PCT levels were available and post-hoc analysis of these patients was performed. Median PCT-concentration was 0.11 ng/ml (IQR 0.06-0.33), with significantly higher values in non-survivors (0.40 ng/ml [0.15-2.06]) vs. survivors (0.10 ng/ml [0.06-0.30], p < 0.001). PCT predicted 30-day mortality (AUC = 0.73) and critical pneumonia (AUC = 0.74) by ROC-curve-analysis. In that subgroup, both PCT and cortisol tended to improve the predictive potential of CRB-65 score for 30-day-mortality, however in binary logistic regression analysis including all three variables, the added predictive value was not significant (p = 0.27 for PCT, p = 0.057 for cortisol). If critical pneumonia was used as dependent variable, only cortisol added significantly and independently of PCT to the prognostic accuracy of CRB-65 (CRB-65 AUC = 0.77; CRB-65 + cortisol AUC = 0.82, p < 0.001). The combined addition of both biomarkers to CRB-65 showed no further improvement.

To assess the prognostic relevance of cortisol in comparison to the suggested minor criteria to identify patients in need of ICU-admission by the ATS/IDSA 2007 guideline [16], we additionally performed a post-hoc analysis on the 425 patients (43%) with all 8 minor criteria (except multilobar infiltrates: no data) available. By including these factors and serum cortisol into a logistic multivariate regression analysis, significant independent prediction of critical pneumonia was seen only for cortisol (OR 1.78 per IQR-increase, p = 0.001) and the three CRB-factors (confusion: OR 5.7, p < 0.001; respiratory rate: OR 1.1, p < 0.001; blood pressure OR 1.02, p = 0.026). After calculating the minor criteria as suggested by the ATS/IDSA-guideline (with a PaO_2 _< 52 mmHg instead of PaO_2_/FiO_2 _< 250 and without multilobar infiltrate), the resulting AUC after ROC curve analysis for predicting critical pneumonia was 0.71 with an optimal cut-off of ≥2 criteria (sensitivity 49%, specificity 88%). Cortisol added significantly to the predictive accuracy of this model in binary regression analysis (combined AUC 0.74, p = 0.003).

## Discussion

The main study result is that increased serum cortisol level is associated with the development of critical disease and increased 30-day-mortality in hospitalised CAP-patients and adds prognostic information independently of the CRB-65 score and the inflammatory biomarkers WBC and CRP. Serum cortisol level improves the predictive power of the CRB-65 score and shows independent prognostic significance within different CRB-65 strata. Its additional predictive value persisted, when cortisol was analysed in combination with urea within the CURB-65 score, PCT or minor criteria suggested by the recently revised ATS/IDSA-guideline [16] to predict ICU-admission.

Our study confirms and expands previous data from three smaller studies, including a total of 401 patients, suggesting cortisol as biomarker for predicting mortality in CAP patients [21-23]. The optimal cut-off for mortality from our data (795 nmol/l) resembles that proposed by these studies (734 nmol/l in [21], 960 nmol/l in [22] and 820 nmol/l in [23]). The main strength of our study is the high number of patients included and the rigorous follow up (only 2/984 missing cases after 30 days), allowing a more accurate evaluation of mortality and critical pneumonia development with a significant number of patients meeting these endpoints.

In univariate analysis, we found the known risk factors age, male sex, nursing home residence, chronic comorbidities, CRB-65 score and the measured inflammatory biomarkers to be associated with both endpoints [26,27]. In concordance with previous studies, severe pneumonia seemed to be less likely to develop in patients with antibiotic pre-treatment [28-30]. In multivariate analysis, only comorbidities, male sex, the CRB-65 score and serum cortisol predicted both outcomes. The addition of urea to the CRB-65 score (CURB65-score) in a large subgroup analysis resulted in improvement of the CRB-65 score, whereas the substitution of age by urea (CURB-score) did not. If cortisol subsequently was added to either score, further prognostic improvement of the same magnitude resulted, suggesting that the prognostic value of cortisol complements that of urea.

The CAPNETZ study also records the cause of death as judged by the treating physician. This subjective statement certainly has a lower value than measured parameters. But, of the 22 patients who died within 30 days despite having an initial cortisol level below the cut-off of 795 nmol/l, only three died from pneumonia as judged by the treating physicians, and two of these three suffered from concomitant bronchial carcinoma.

Mortality prediction might not be optimal to identify patients at risk for critical disease manifestations such as respiratory insufficiency or septic shock. These carry a high demand on and benefit of intensified management strategies, which makes their early detection crucial. On the other hand, CAP frequently occurs in elderly and multimorbid patients. Here mortality does not always reflect "dying from pneumonia" but rather "dying with pneumonia", and treatment restrictions might bias this endpoint further. By the inclusion of the predefined composite endpoint of critical pneumonia, our study provides even more accurate pneumonia-specific risk prediction than by mortality prediction alone. This dichotomy of endpoints is reflected by our data: Whereas mortality was predicted by concomitant malignancy, chronic renal failure and high age, critical pneumonia was predicted by higher CRP levels indicating systemic inflammation. CRB-65-score, congestive heart failure, male sex and serum cortisol as only biomarker independently predicted both endpoints, confirming the prognostic relevance of cortisol for CAP-related outcomes.

Rapid and accurate identification of hospitalised CAP patients at risk of a critical disease course is a yet unmet need in CAP management. Several scores have been evaluated for predicting ICU-admission. The "classical" risk prediction tools, the CRB-65- and PSI-scores perform well in identifying patients with a low mortality risk, but are poor in predicting ICU-admission [14,20]. Recommended tools for this purpose are the modified ATS-rule [15], consisting of two major criteria (requirement of mechanical ventilation, presence of septic shock) and three minor criteria (systolic blood pressure < 90 mmHg, multilobar involvement, PaO2/FiO2 < 250), and the newer ATS/IDSA-rule [16], which added 6 more minor criteria (respiratory rate > 30/min; confusion; blood urea nitrogen > 20 mg/dl; leukopenia; thrombocytopenia; hypothermia), thus incorporating all "CRB"-criteria. If these factors were included in a multivariate analysis with serum cortisol in our study, only cortisol and the three CRB-criteria provided independent predictive power for critical pneumonia, suggesting potential improvement of this score by complementing or substituting some of these criteria by cortisol.

To account for the heterogeneous causes for deterioration in CAP, optimal risk prediction probably should incorporate clinical criteria plus biomarkers reflecting inflammatory, cardiovascular and other mechanisms of disease progression. Accordingly, although inflammatory markers like CRP [13] and PCT [6,31] are associated with mortality in CAP, their predictive accuracy does not allow high risk prediction by themselves. Cardiovascular biomarkers like NT-proBNP [11], copeptin, pro-endothelin and MRpro-ANP [9,32] and others like d-dimer [12] and proADM [10,33] also predict poor prognosis in CAP.

Cortisol is a biomarker reflecting additional mechanisms involved in critical disease development like stress response and immunomodulatory regulation of inflammatory processes [34]. Physiologically, acute stress like severe illness leads to an activation of the hypothalamic-pituitary-adrenal axis which protects the organism against excessive inflammatory responses [35]. In severe sepsis and septic shock, the increase of serum cortisol levels parallels the severity of infection and prognosis of patients [36]. This makes cortisol an attractive biomarker reflecting acute patterns of critical disease and disease progression. Recently, three small studies showed that cortisol correlates to CAP severity and predicts mortality in CAP-patients. Data from our group additionally demonstrated, that clinical instability after 72 h, reflecting course and treatment response in CAP, was predicted by admission cortisol levels [21]. Our present study data add to that evidence by demonstrating prediction of critical pneumonia development independently of known clinical risk factors and inflammatory biomarkers. However, before cortisol measurement can be recommended for clinical routine use as biomarker in CAP, a prospective interventional trial is necessary to prove its accuracy and cost-effectiveness in comparison to evaluated clinical scores and competitive biomarkers for predicting patients benefiting from intensified treatment and monitoring strategies.

Recently there have been conflicting reports on benefits and risks of steroid treatment in CAP [37,38]. Given the accumulating evidence of the association of high cortisol levels with worse outcome in CAP, which is confirmed by the present data, the rationale of steroid treatment for this condition should be questioned. Whether the association of high cortisol and poor prognosis reflects adrenal regulation because of more severe CAP or adrenal dysregulation resulting in a complicated disease course cannot be concluded from our data and deserves further study.

Several limitations of our study have to be mentioned:

First, we were not able to correct for concomitant steroid medication, as this data were not documented. Systemic steroid medication of ≥ 20 mg prednisolone equivalent per day for more than 14 days excludes participation in CAPNETZ. In order to control for potential effects of steroid co-medication, which probably affect patients with chronic respiratory disease in particular, analysis after exclusion of all patients with chronic respiratory disease was performed and no relevant change of the diagnostic properties of cortisol could be detected.

Second, as blood samples were taken at time of first contact, controlling for the time point of blood sampling could not be done. Cortisol exhibits diurnal concentration changes; however, during infectious diseases the circadian pattern is often lost [39].

Third, we did not test for adrenal insufficiency based on the response to injection of synthetic adrenocorticotropin. However previous data show a very low rate of adrenal insufficiency in patients with CAP in the absence of septic shock [21], and the association of mortality with high cortisol levels seems to contradict any major prognostic influence.

Thus, deviation of our study results by concomitant steroid medication, adrenal insufficiency or different blood sampling time points cannot be ruled out. These limitations also might account for the slightly lower predictive performance of cortisol shown in our study when compared to some of the previous data (AUC for mortality in our data 0.70; 0.76 in [22], 0.65 in [23], 0.83 in [21]). Standardised blood collection at the same daytime in all patients and rigorous exclusion of oral steroid co-medication would most probably have resulted in a higher accuracy but does not reflect daily clinical practice. Therefore, this lack of standardisation might also be interpreted in favour of the robustness of the association between serum cortisol and CAP outcomes.

Finally, a comparison with other non-inflammatory biomarkers of CAP prognosis or new risk scores recently evaluated to predict ICU admission in CAP like the SMART-COP score [40] is not available from our data; and comparison with urea, PCT and the ATS/IDSA-minor criteria have been calculated in post-hoc analyses with data not complete for all patients.

## Conclusions

In conclusion, cortisol predicts mortality and critical disease in hospitalised CAP-patients independently of clinical factors and inflammatory biomarkers. Recognizing the need for early identification of patients requiring intensive management strategies and the complex mechanisms involved in critical disease development in CAP, cortisol represents a promising biomarker for high risk prediction. In order to implement its use into clinical practice, the optimal combination of clinical factors and biomarkers for high risk prediction in hospitalised CAP should be evaluated in a large prospective trial. Ideally, this would include an interventional design, assessing the guidance of more intensive monitoring and management strategies based on a combined risk stratification tool consisting of clinical factors and biomarkers like cortisol.

## Competing interests

The authors declare that they have no competing interests.

## Authors' contributions

Author contributions: conception, hypothesis and design of the study (MK, MWP, GH); acquisition of data (MK, MWP, GR, HS, RB, NS); analysis and interpretation (MK, PM); substantial involvement in the writing and/or revision of the article (MK, MWP, PM, GR, HS, RB, GH, NS). All authors read and approved the final manuscript.

## Pre-publication history

The pre-publication history for this paper can be accessed here:

http://www.biomedcentral.com/1471-2334/12/90/prepub
